# Similar but Different: Dynamic Social Network Analysis Highlights Fundamental Differences between the Fission-Fusion Societies of Two Equid Species, the Onager and Grevy’s Zebra

**DOI:** 10.1371/journal.pone.0138645

**Published:** 2015-10-21

**Authors:** Daniel I. Rubenstein, Siva R. Sundaresan, Ilya R. Fischhoff, Chayant Tantipathananandh, Tanya Y. Berger-Wolf

**Affiliations:** 1 Ecology and Evolutionary Biology, Princeton University, Princeton, NJ, United States of America; 2 Jackson Hole Conservation Alliance, Jackson, WY, United States of America; 3 University Corporation for Atmospheric Research, Washington, DC, United States of America; 4 Mpala Research Centre, P.O. Box 555, Nanyuki, Kenya; 5 Google Inc., Mountain View, CA, United States of America; 6 Computer Science, University of Illinois at Chicago, Chicago, IL, United States of America; Tianjin University, CHINA

## Abstract

Understanding why animal societies take on the form that they do has benefited from insights gained by applying social network analysis to patterns of individual associations. Such analyses typically aggregate data over long time periods even though most selective forces that shape sociality have strong temporal elements. By explicitly incorporating the temporal signal in social interaction data we re-examine the network dynamics of the social systems of the evolutionarily closely-related Grevy’s zebras and wild asses that show broadly similar social organizations. By identifying dynamic communities, previously hidden differences emerge: Grevy’s zebras show more modularity than wild asses and in wild asses most communities consist of solitary individuals; and in Grevy’s zebras, lactating females show a greater propensity to switch communities than non-lactating females and males. Both patterns were missed by static network analyses and in general, adding a temporal dimension provides insights into differences associated with the size and persistence of communities as well as the frequency and synchrony of their formation. Dynamic network analysis provides insights into the functional significance of these social differences and highlights the way dynamic community analysis can be applied to other species.

## Introduction

Social animals interact in diverse ways when forming groups, choosing mates, competing or cooperating and exchanging information. While some encounters are casual, others are long lasting and come in a variety of forms. Ultimately this variation in sociality emerges from differences in the physical and social environments animals inhabit [1, 2]. Yet, societies that are sometimes categorized as similar, often turn out to be deeply different. Understanding why variations on a common theme appear and what functions these differences serve, remains elusive. Social network analysis, however, is helping provide some clues by showing that even evolutionarily closed related species differ in the diversity, strength and reach of the relationships, but sheds little light on the adaptive significance of these differences [3–6]. Since both the environmental forces that select for social differences, as well as the comings and goings of individuals that characterize all fission-fusion species are spatio-temporal in nature, any understanding of why variations in sociality emerge will require coupling social and environmental temporal dynamics into network analyses. Although individuals in fission-fusion societies frequently change groups, they retain some level of persistent social affiliations, thus forming communities. But variation in individual movement synchrony will lead to differences in community persistence and composition. Identifying which communities individuals belong to matters since balancing cooperation and competition, being altruistic and identifying and ostracizing cheats are all facilitated by repeated interactions that vary in frequency depending on consanguinity, familiarity and the consequences of failing to solve problems posed by nature. All of these various aspects of interactions, from how they are defined to the various types and scales of interactions, matter for the conclusions one might draw from the analysis. Here we focus on the temporal dimension of network analysis and we apply algorithms [7–10] that identify dynamic communities. We extract a series of metrics that characterize species-specific differences in individual decision- making and their social consequences and offer insights into the functional significance of differences in sociality.

One of the best examples of evolutionarily closely related species showing variants on the common fission-fusion societal theme is the Grevy’s zebra (*Equus grevyi*) of central Kenya and the Asiatic wild ass (*Equus hemionus*), the onager of the little Rhan of Kuch, India. Traditional “static” network analysis has shown for populations of similar size [3] that Grevy’s zebras exhibit more connected components than onagers, the size of these components is on average smaller and less variable than those of onagers, that average cluster coefficient is larger than onagers and average path length is smaller than onagers. After applying dynamic network analysis the differences are magnified and reveal how, and possibly why, these patterns come about.

## Methods

We use dynamic social network analysis to quantify similarity and difference in sociality of two equid species, Grevy’s zebra and onager.

### 0.1 Static and Dynamic Networks

**Fig 1:**
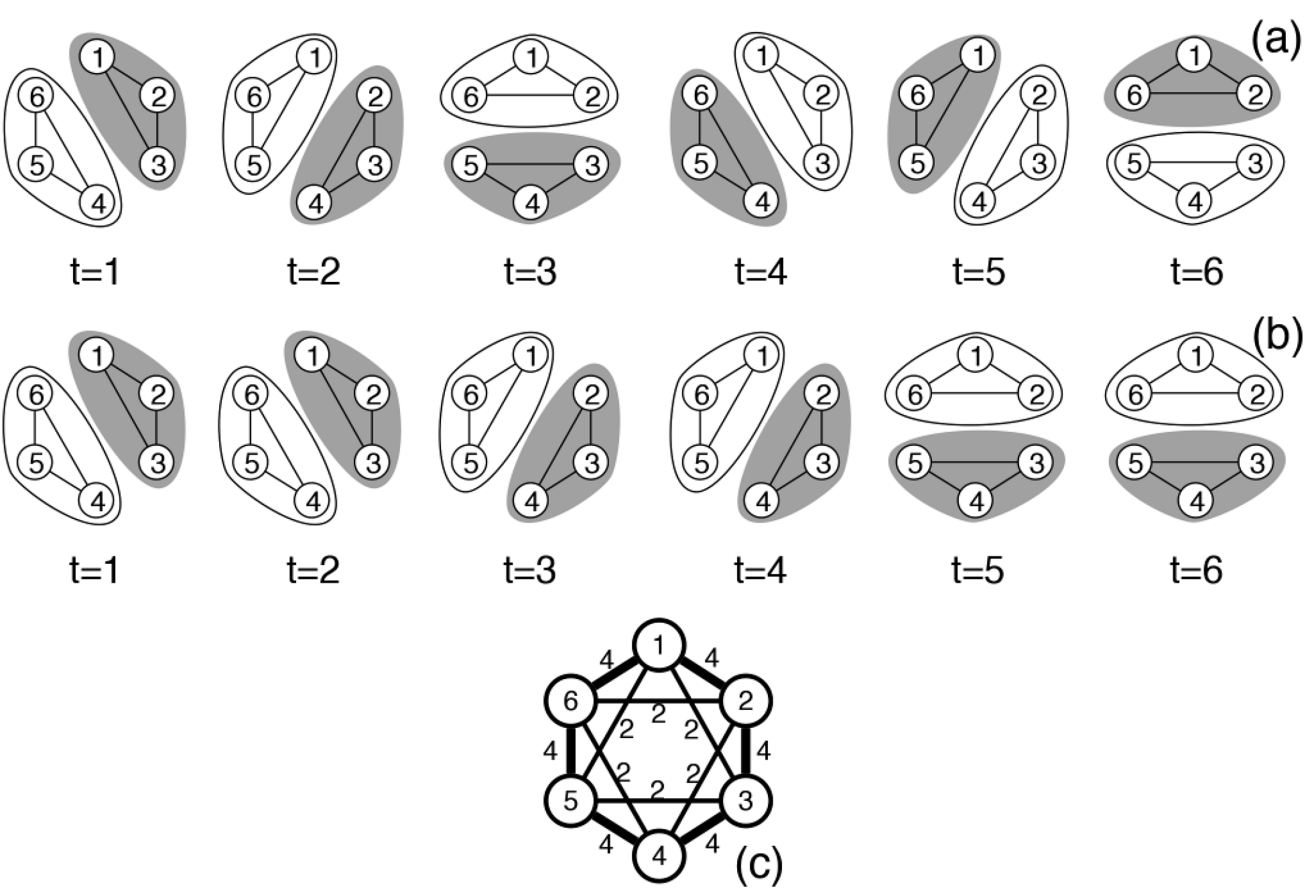
Examples of two different dynamic networks, (a) and (b), that lead to the same static network (c) when aggregated over time.

In a typical social network individuals are represented by nodes, which are linked by edges if they have interacted. An “interaction” may be direct, such as grooming, fighting, having a phone conversation, or sending an email. Alternatively, an “interaction” can be an inferred indirect relationship, such as correlation of a set of features (buying habits), similarity in the trajectory, or represent a simple spatial proximity of being close enough for long enough (but see [11] for an excellent discussion on the appropriateness of proximity networks in general and the pitfalls of social network analysis in animal studies and beyond). These different types (modalities) of interactions can be separated into different networks for explicit comparison [12], combined into one multimodal (also known as heterogeneous, multi-layered, multiplex, multi-relational) network [13–19], or simply represented as one network, discarding the information about the interaction types. The edges may have an associated weight representing the frequency, strength, or probability of interactions. Most conventional network methods aggregate all observed interactions over a period of time and build one network to represent all interactions over that time period [20–23]. For example, in Fig 1(c) we show a hypothetical network that represents all the interactions that have occurred at any point among a set of individuals.

However, in many situations, interactions among entities change over time. Many different scenarios may result in the same aggregate network view. Fig 1(a) and 1(b) illustrate different time series of six individuals interacting over six time steps. Each series of interactions would have the same aggregate representation of Fig 1(c).

We use a *dynamic network* framework [24–30] as a convenient representation for explicitly modeling temporal changes in interactions. In a dynamic network, interactions are typically represented by a time-series of static networks, each network corresponding to interactions aggregated over a small time period, such as a day or hour. (An alternative representation of dynamic networks as a stream of edges is often employed in large communication networks with explicitly defined interactions, such as email and cellphone networks.) The issue of time step size is a complicated one and only now is beginning to be addressed in a principled and rigorous way [31].

Typically, we use various network measures to understand how much overall interaction happens in the network (density), how gregarious various individuals are (degree distributions), how connected the network is (path length distributions) and how modular, or clustered, the interactions are (communities). The latter, communities, are clustered sets of individuals “among whom there are relatively strong, direct, intense, frequent, or positive ties” [20]. Individuals in communities typically have higher clustering coefficients (the number of friends who are friends themselves), or other measure of network proximity and density, than those outside. All these measures have been extended to dynamic networks [10, 30, 32, 33], and we present a summary of the definitions in Table 1.

**Table 1:** Static and dynamic network measure of Grevy’s zebra and onagers.

Metric	Grevy’s	Onager
Number of individuals	27	29
Number of time steps	44	82
Number of days	58	163
Number of groups	149	350
Density	0.30	0.36
Dynamic density	0.52	0.24
Average shortest path	1.8	1.7
Average shortest temporal path	4.8	7.5
Diameter	4	3
Dynamic diameter	36	74
Clustering coefficient	0.9	0.7
Dynamic clustering coefficient	0.1	0.03

Table showing comparative data characteristics and metrics calculated from static and dynamic networks for the two species

### 0.2 Communities in Networks

In network literature and social sciences there is an implicit assumption that groups of individuals (or entities, such as protein molecules) that interact more densely among themselves share a commonality of reason, or purpose, and functionality [34–36]. A “community” in a social network is an abstraction that allows to group nodes based on interaction data. Uncovering these groups, or communities, is, however, the first step in understanding the fundamental causes and consequences of (social) interactions. Similarly, the focus of many sociobiological studies is to identify the causes, consequences and functionality of associations [37–39]. The groups with which individuals associate vary in size and cohesion because of the variations in the nature, context, and frequency of interactions, as well as the relationships they produce. Biases emerge in the subset of associating individuals because kinship and reciprocity generate different patterns of cost-benefit tradeoffs. The aim of community inference methods is to group nodes into clusters that function in similar ways, as is implied by the closer interactions. Understanding these similarities that define a community is a first step towards understanding the adaptive value, and hence evolutionary functionality, of particular communities and the underlying relationships that produce them.

### 0.3 Static network community identification

From a network perspective, communities are loosely defined as regions of a network with dense connections within the region, relative to its surroundings. In other words, a community is a set of individuals more closely affiliated with each other than with members of other communities. A number of community identification methods have been historically used in sociology and biology. With the recent increase in the size of network datasets, there has been an explosion in computational community identification tools [36, 40–43]. As mentioned above, a major disadvantage of these methods is that the temporal component of interactions is discarded.

The concept of *dynamic* communities overcomes this problem. A dynamic community is a collection of individuals who interact more frequently, contiguously and persistently among themselves than with other individuals [10]. We show that the concept of dynamic communities uncovers heretofore hidden patterns and processes in networks using our two equid species as examples. We argue that our method is widely relevant and could provide greater insights into animal sociality, especially in cases where interactions vary with time, as occurs widely across species. By extending the traditional static view of a community as a set of individuals to now include the individuals and their interactions over a time period, we believe that it is possible to better understand structure and functioning of animal societies (including humans).

### 0.4 Identifying dynamic communities

The motivation for identifying communities and the assumption underlying community inference methods is that communities are latent structures that are manifested by the observed interactions. Computational dynamic communities inference methods take two conceptual approaches: temporally stringing static communities identified in each time step [29, 44–48] or clustering interactions over time to optimize some objective such as relative temporal density within communities versus outside or to minimize the change in community membership [16, 49–55].

CommDy is one of the more prominent dynamic community inference methods that combines the two conceptual views and is grounded in the biological and behavioral foundations of social interactions [7, 8, 10]. Given a dynamic network, CommDy uses (combinatorial) optimization to group nodes into communities in a way that minimizes the overall changes in the inferred community affiliation of individuals. CommDy is unique in that it explicitly allows for fluid community membership over time and infers (rather than just describes) communities without requiring any knowledge of the initial state. The fundamental assumption of CommDy is that communities tend to change gradually over time [?, 56–59], as opposed to assembling or disbanding spontaneously. This view is motivated by the intuitive understanding of a community as a body which exists for a certain time period, during which it has consistent membership and welcomes few outsiders, while allowing for fluidity in group membership. The method leverages this notion of purposeful fluidity and persistence, and axiomatizes it in a way that explicitly draws a connection between latent community structure and observed interactions. Based on this axiomatization, a combinatorial optimization problem is formulated. CommDy assumes an input of a dynamic (social) network, which, for its purposes, is a time series of static networks. CommDy also assumes that the nodes in each of these static networks have been grouped into communities local to each time step. These groupings may come directly from observations (meetings of people, sightings of groups of animals, club membership), or inferred from the network using any of the static community inference methods [61–63]. These grouping instances are seen by CommDy as manifestations of the latent underlying dynamic community structure. Nodes grouped together at any given time may be either members of the same community at the moment or include temporary visitors from other communities. And while members can switch community affiliations over time or visit groupings of other communities, these switches and visits, as well as absences from groupings of their community have a social cost associated with the actions based on assumptions derived from social network theory [20, 59].

These costs are grounded in biology. In animal societies, individuals incur real social costs when their behavior deviates from these assumptions. We can observe physiological and other costs when an individual permanently joins a new group, when it visits a different group, or when it is left behind by their mates. In equids, for example, individuals changing groups often incur costs. Female horses are typically harassed by residents when changing groups [64] and lactating Grevy’s zebra females moving among territorial males receive higher levels of harassment than females remaining on the territory of one male [3].

The particular cost settings are part of the input to CommDy, but it is only their relative values that are relevant and those can be typically assessed for any given network. Moreover, stability and sensitivity analysis can be performed to evaluate the impact of different cost settings on the resulting community structure.

**Fig 2:**
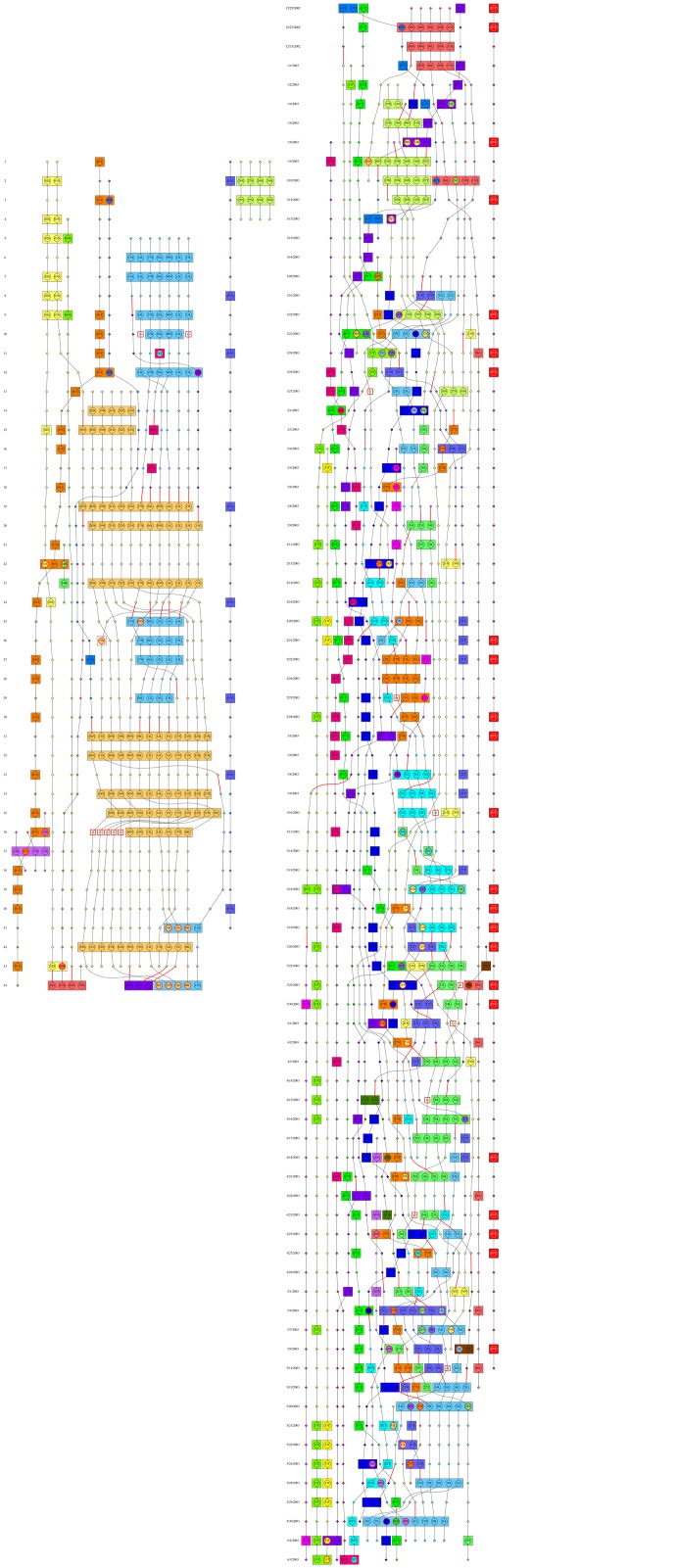
Inferred dynamic communities of (a) Grevy’s zebra and (b) onagers with all costs set equal to 1.

Thus, given a dynamic network, the cost settings, and a static community structure at every time step, CommDy finds the most parsimonious dynamic community structure that minimizes the overall cost of switches, visits and absences by all the nodes. The algorithm proceeds by constructing an auxiliary group graph. The nodes are groups or the inferred static communities in every time step and the edges connect communities in two consecutive time steps, weighted by the relative number of common individuals (we use Jaccard similarity [65], the size of the intersection divided by the size of the union). CommDy then finds an optimal weighted matching [66] on the group graph and uses dynamic programming [67] to find the optimal individual community assignment that minimizes the costs, given the group community structure. An equivalent view of CommDy is as the maximum likelihood fit of the following model to the given time series of grouped networks data: all groups within a time step are manifestations of some community, each individual starts out as a member of some community and at each time step it has a (less than 0.5) probability of switching its community affiliation and a (less than 0.5) probability of visiting a group which does not belong to its community. This model is a natural dynamic extension of a standard block-mixture model for static networks [68, 69]. Regardless of whether the maximum likelihood or parsimony formulation is used, the resulting optimization problem itself is computationally intractable (NP complete) and CommDy is an approximation algorithm, the first (and to date the only) with a provable approximation guarantee for dynamic communities inference [8], which allows near-optimal solutions in short time (and is optimal in practice). What emerges out of this analysis is a flow diagram of a dynamic community structure (see Fig 2). For a full mathematical description of the method we refer the reader to our earlier book chapter [10].

### 0.5 Dynamic community interpretation

Dynamic community identification methods find the optimal (with respect to the specific social costs) community structure of the network. Each community is represented by a different color. The color of a node at each time step, therefore, represents its individual’s community affiliation at that time. Each group is the color of the community it represents. Switching an individuals’ community affiliation corresponds to that individual’s node being a different color from one time step to another. An individual visiting another community is going to be a different color from the group it is observed at that time step. Once such a coloring has been found, each community is the set of groups of a particular color over time. The community structure is then the collection of all communities. Note, that we explicitly allow a community’s membership to change or evolve over time.

Once the optimum community structure is determined, several metrics can be computed to describe that structure of a dataset (Table 2). The simplest measures count the number of communities, their sizes and their duration (*span*). In addition, we can identify the core versus peripheral members of communities (*visiting cost, switching cost*, and *peer coordination*) by looking at the visiting and switching costs that individuals accumulate. From a community perspective, *homogeneity* is the ratio of visitors versus members present in that community. The more homogeneous a community is, the more plausible it is that the coming together of particular individuals is not accidental and is driven by some similarly perceived environmental force. Of course, other metrics can be computed as needed for various analyses, in addition to the ones listed in Table 2 as examples. Finally, we can use these metrics to compare community structures of different datasets or of the same dataset at different times. The list of dynamic metrics used in our analyses is highlighted in Table 2.

**Table 2:** Dynamic community metrics.

**Group attributes**	
Size	The number of individuals, both community members and visitors (but not absents), in a group.
Homogeneity	The fraction of group members who have the same community affiliation as the group (total number of individuals who have the same community membership as their current group, divided by the group size).
**Community attributes**	
Span	Total span of time steps that a community exists: the last time step minus the first time step of the community’s existence.
Apparancy	The fraction of time steps a community is present over its span.
Size	The number of individuals (members and absents but not visitors) affiliated with the community, averaged over the number of time steps a community is present.
**Individual attributes**	
*Switching cost*	*The number of community switches made by an individual in a population (normalized by the number of time steps an individual is observed).*
*Visiting cost*	*The number of visits made by an individual to another community in a population (normalized by the number of time steps an individual is observed).*
Absence cost	The number of absences of an individual from a community in a population (normalized by the number of time steps an individual is observed).
*Community stay (Membership continuity)*	*The average number of consecutive time steps an individual stays affiliated with the same community over the time steps it is observed.*
Peer	Peers of an individual are other members of the same group that share the same community identity. (Note, that they do not need to share the group community identity, just each others?.) We compute the average number of peers of an individual over the observed period.
*Peer coordination*	*At each time step, peer coordination is the fraction of the current peers that were peers in the previous time step. We compute the normalized peer coordination of an individual over the observation period.*
Group size	Average size of the groups of which an individual is a member.
*Group homogeneity*	*Average homogeneity of the groups of which an individual is a member.*
*Community size*	*Average size of the communities with which an individual is affiliated.*
*Community span*	*Average span of the communities with which an individual is affiliated.*
Community apparancy	Average apparancy of the communities with which an individual is affiliated.

Dynamic community metrics definition for a group and an individual. Variables used in the analysis here are italicized.

For each individual and community these measures define a multidimensional statistical feature space that can be then further analyzed using standard statistical and machine learning techniques, such correlation, principle component analysis (linear or not), classification, or Bayesian and likelihood inference.

### 0.6 Data

We made group membership observations of Grevy’s zebra in Kenya and Asiatic onager in India, previously described in [3]. For both populations, the data were collected on a daily basis and during each day we searched the study area for herds (groups) by driving a predetermined route. Upon encountering a herd, we recorded the identity of each individual in the group using unique identifying marks. Repeated spatial sampling is the traditional and still the most prevalent way to collect association data on animals [23]. The Grevy’s zebra dataset includes 28 individuals observed over 44 daily samplings (time steps) between June and August of 2002. The onager dataset has 29 individuals, observed over 82 daily samplings (time steps) between January and May 2003. There were no biological tissue samples collected and only observational data was recorded about the animals. The activities were allowed under the research permit NACOSTI/P/14/1003/1628 and reviewed and approved by the Princeton IACUC 1835-13 for Wildlife Wireless Tracking.

**Fig 3:**
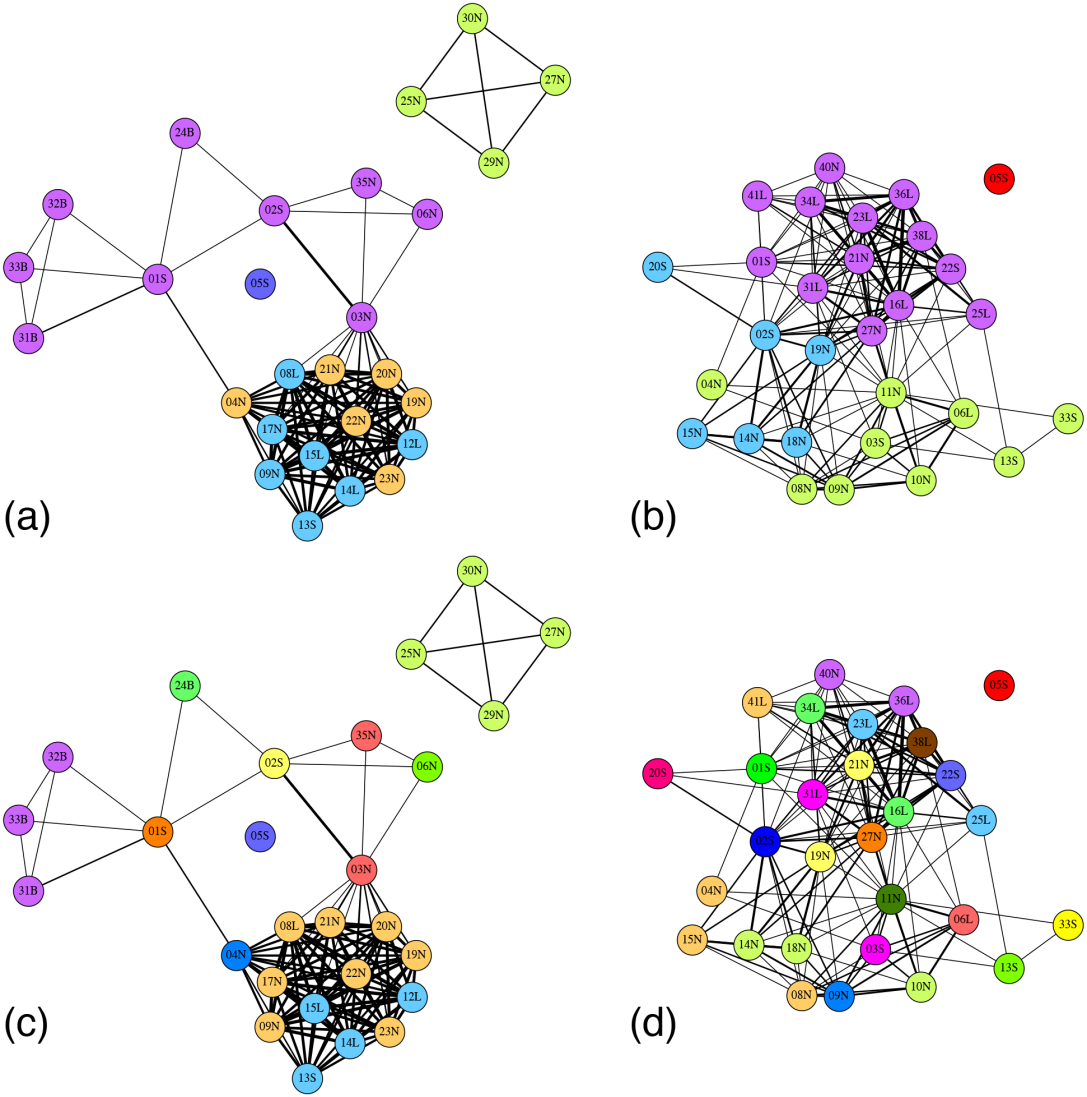
Static communities of Grevy’s zebra and onagers detected using Louvain algorithm ((a) and (b)) and the superimposed dynamic communities, where each node is colored by the majority color of its dynamic communities ((c) and (d)).

We form social networks where each individual is a node and two individuals are connected if they have been observed together (in the same group) at some time. While, as mentioned, proximity networks can be problematic in general [11] it the appropriate definition of an edge for the current study which aims to understand the causes and consequences of animal association in groups. For a dynamic network, the time step duration is one day, which is determined by the field sampling regime. For a static network the associations are aggregated into weighted edges over the entire time line. Fig 2 shows the dynamic networks for the two species, Fig 3 shows the static aggregated networks, and Table 1 shows the basic static and dynamic network measures for the two species.

We use Louvain algorithm [62] to infer static communities in the aggregate static network. Given the dynamic network, we use CommDy to infer the dynamic community structure, using the observed groupings in each time step as the basis of inference. We use equal values settings for switching, visiting, and absence. We have performed analysis for different relative cost settings. However, since the resulting community structures were quite similar and the subsequent analysis showed no statistically significant differences, we use the simple equal value cost settings for presentation and the resulting conclusions. See supporting information for the details of the analysis (S1 File). We then compute all the measures on the dynamic community structures and use linear Principle Component Analysis (PCA) to identify the community structure measures that are significant and meaningful in recognizing the differences between the two species, sexes, and animal phenotypes based on reproductive status (lactating females, non-lactating females, territorial males). For each PCA we examined the loadings on the components and ran a t-test [70, 71] to measure the statistical significance of the difference where it was detected. In addition, we have performed cross-validation using radial SVM [72] to model species based on dynamic community statistics. In each of the 99 random simulations, we randomly picked 10% of the individuals from each species to be reserved as a testing set. We trained the radial SVM on the remaining 90% of the data set, and useed it to predict the species of the reserved 10% testing data set. The radial SVM correctly classified the species for 63.96% of the time. However, PCA analysis was sufficient and provided the same information as the more advanced statistical techniques and, thus, we present only the PCA results here.

## Results

The static network metrics in Table 1 reveal some small differences in the sociality of the species. The dynamic metrics amplify them. Grevy’s zebras dynamic density is almost twice as high as the static and almost four times the dynamic cliquishness of onagers, and both the average shortest temporal path and dynamic diameter are half that of onagers. This suggests that at any point in time the connections among individuals Grevy’s zebra are more numerous and more direct.

Fig 3 shows the aggregate static networks of the two species and the inferred static communities.

Fig 2 shows the dynamic community colorings at each time step inferred by CommDy algorithm for Grevy’s zebras (2(a)) and onagers (2(b)) with equal costs assigned to switching, visiting and being absent (see Figs A, D, and G in S1 File for other cost settings). By inspection, Grevy’s zebras show long consistent patterns of the merging and splitting of a few homogeneous communities, whereas the onagers show much mixing among many small communities, often consisting of only a single individual. Because of familiarity with static network graphs we superimpose the dynamic community colorings of the Grevy’s zebra and onager on the static networks (Fig 3(c) and 3(d) presented in [3] (see Figs B, E, and H in S1 File for other cost settings). Each individual’s coloring corresponds to the community of which it was a member for the majority of sightings. This visualization confirms the impression from the dynamic community timelines that onager society is a well-mixed assemblage of many independent individuals. Conversely, this superimposition of the dynamic community identities on the static network reveals previously hidden structure. First, the majority of the largest static community in Grevy’s zebras is comprised of two dynamic communities–orange and blue. Interestingly, the orange community consists of non-lactating females whereas the blue community consists of lactating females, both associated with one territorial breeding male.

While visualizations can provide initial insights into social structure, quantitative metrics identify those structural difference that are biologicaly significant and can help unravel the selective forces and adaptive value of specific patterns of sociality. Seven metrics listed in Table 2 proved informative for showing sex and reproductive status differences in these two equid species.

**Fig 4:**
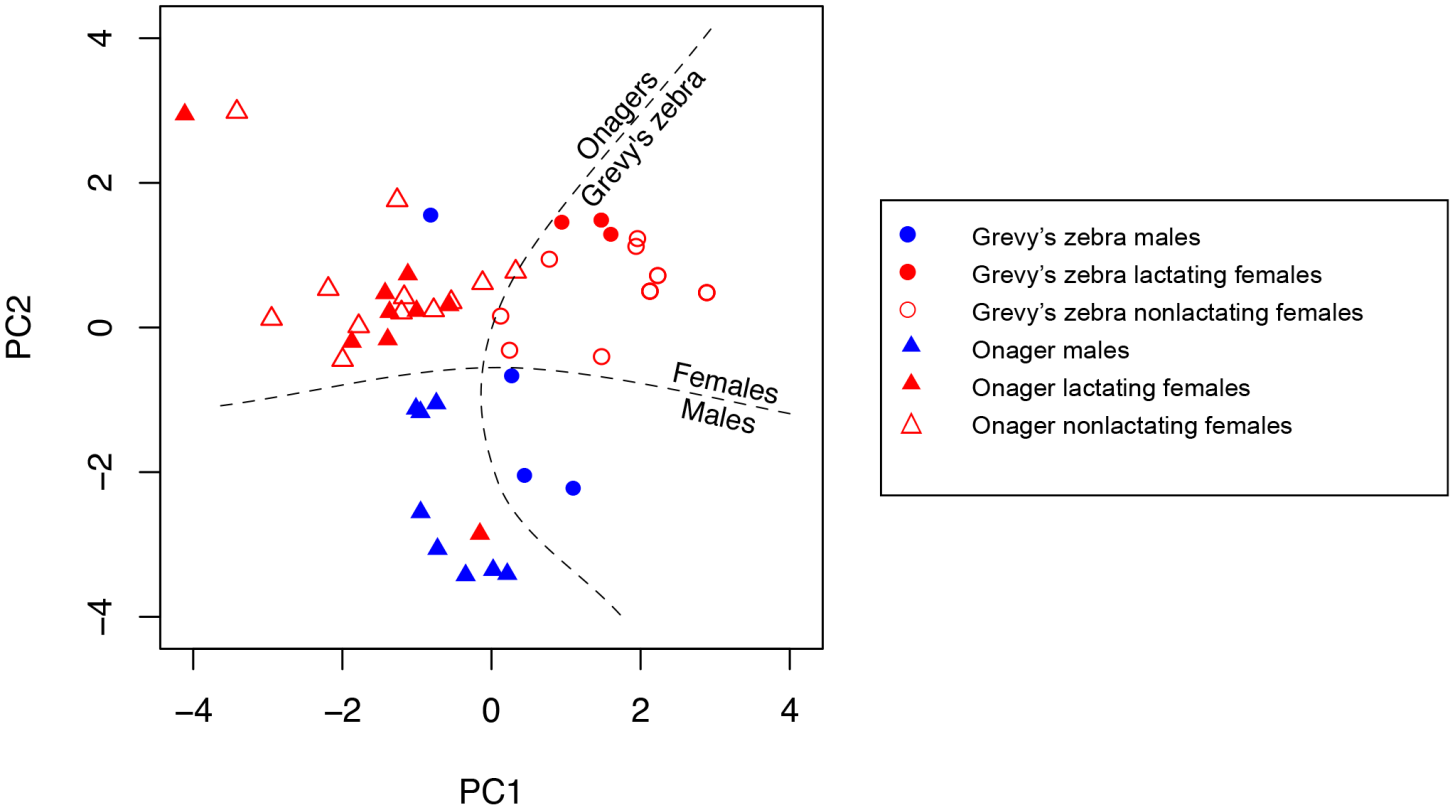
Projection onto the first two principle components of the dynamic communities metrics of all the individuals in both Grevy’s zebra and onagers.

Principle Component Analysis showed that the first two components account for 64% and 35.6%, respectively, of the variance in the community metrics of all the individual equids. The loadings on the variables on the first two components are 0.9 for community stay and 0.46 for community span for component one (PC1), −0.46 for community stay and 0.9 for community span for PC2. When all the individual equids are placed in the statistical feature space projected on to the first two principle components, strong separation by sex and species emerge (see the biplot of the first two components in Fig 4 and Figs C, F, and I in S1 File for additional cost settings).

**Fig 5:**
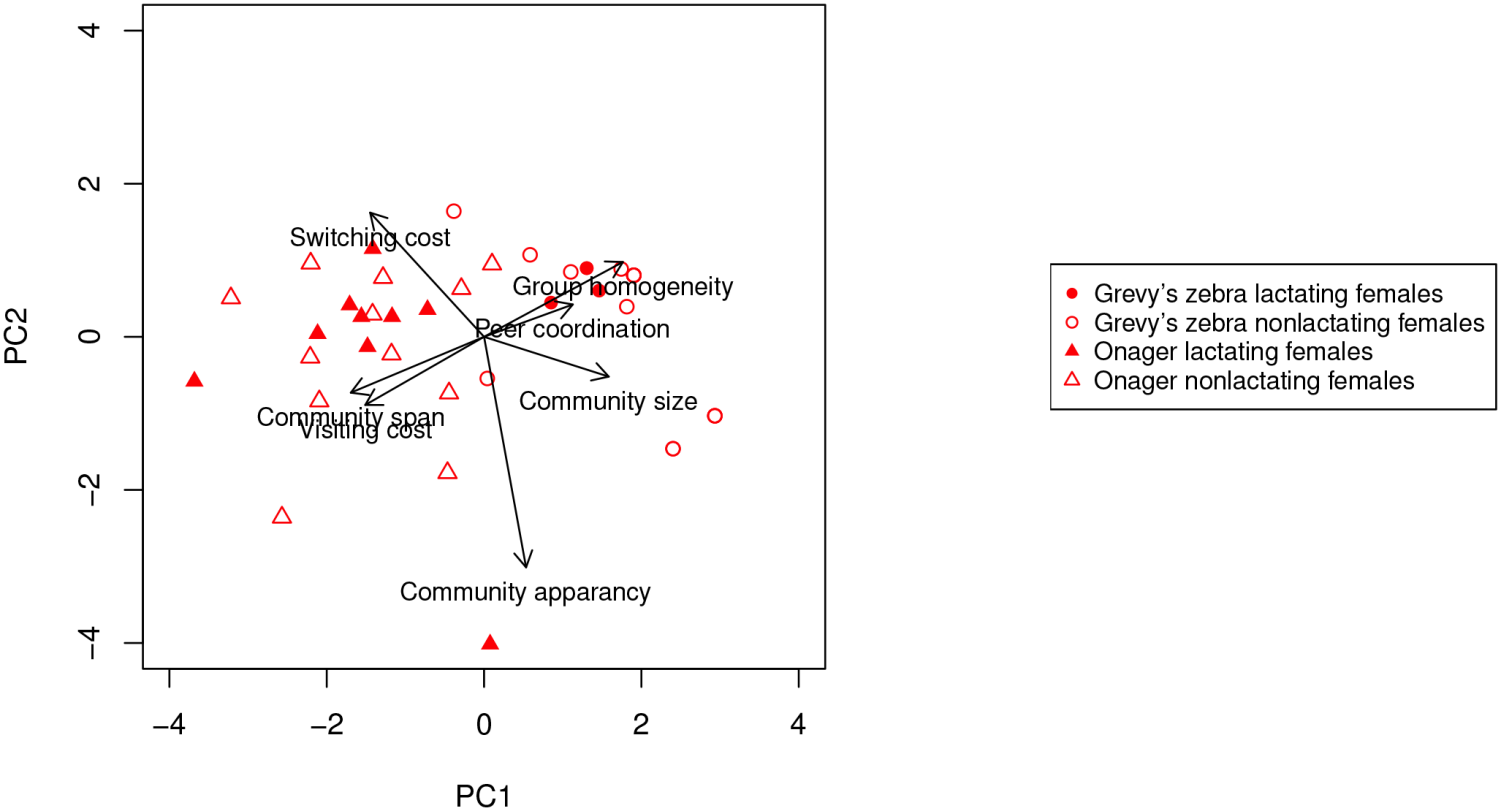
Projection onto the first two principle components of the dynamic communities metrics of all the females in both Grevy’s zebra and onagers.

In order to determine the factors contributing to these differences, we further explore which dynamic community metrics are significant in producing these separations. As Fig 5 illustrates, in females, the main separation is aligned with the dynamic metrics of community span and visiting cost (positive for onagers) and group homogeneity and peer coordination (positive for Grevy’s zebras). The two-sample t-test ratio between the species is 10.1 for PC1 (*p* < 0.0001) and 1.23 for PC2 (*p* = 0.25). Biologically, this indicates that while onager females visit other communities often, they retain their own long-lived community identity. Interestingly, because onager females also show a strong negative loading on community size, their somewhat persistent communities are small, often communities of one! Grevy’s zebra females, however, form relatively large homogenous cohesive groups.

**Fig 6:**
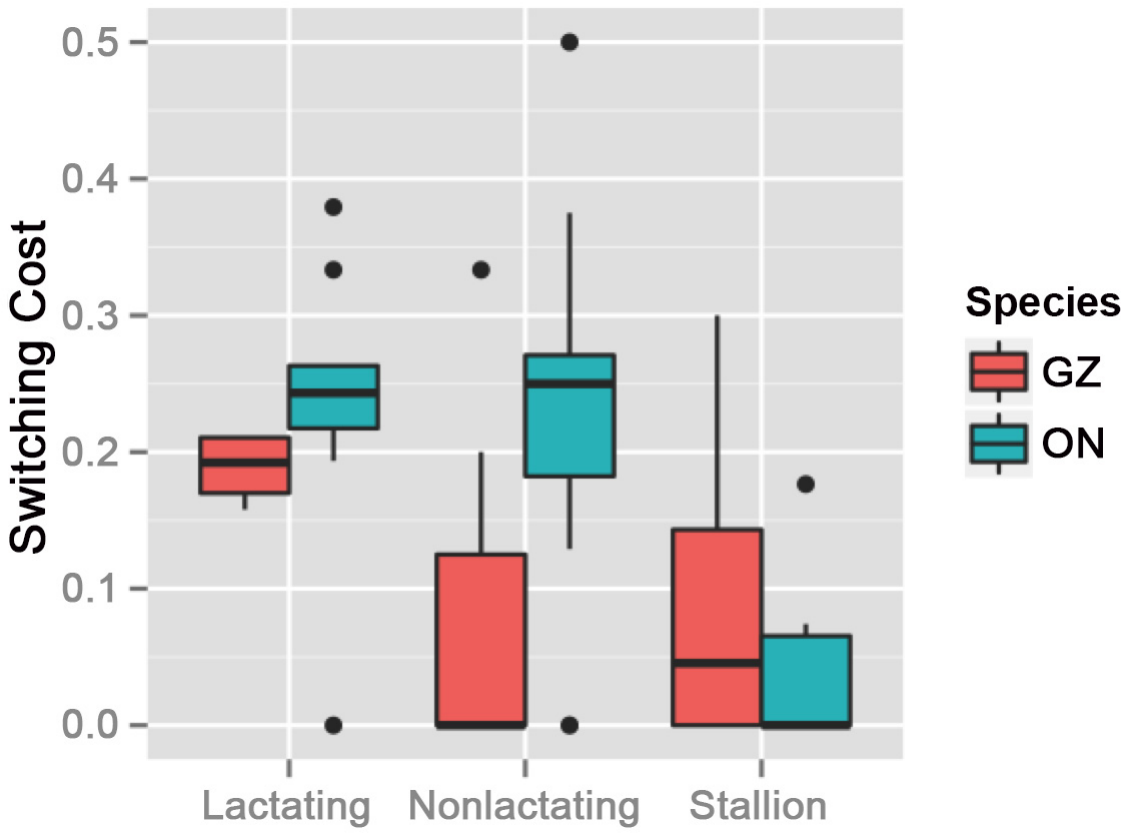
Switching costs of both Grevy’s (red) and onagers (blue), by reproductive status. The line within the box is the mean value, the box encompasses the 1st quadrille from the mean, the whiskers denote the 3rd quadrille, and the points are at 5% and 95%.

Probing deeper into the nature of female community structure (Fig 6) Grevy’s zebras, but not onagers, show statistically significant differences in their switching cost in relation to their reproductive state. While all onager females, irrespective of reproductive state, reveal a higher (but still low) propensity to switch communities than Grevy’s zebras, non-lactating Grevy’s zebra females tend not to switch communities at all (Fisher exact test *p* = 0.0015). Only lactating Grevy’s zebras change community identity when groups merge, yet, still, their switching is low and has almost no variance.

**Fig 7:**
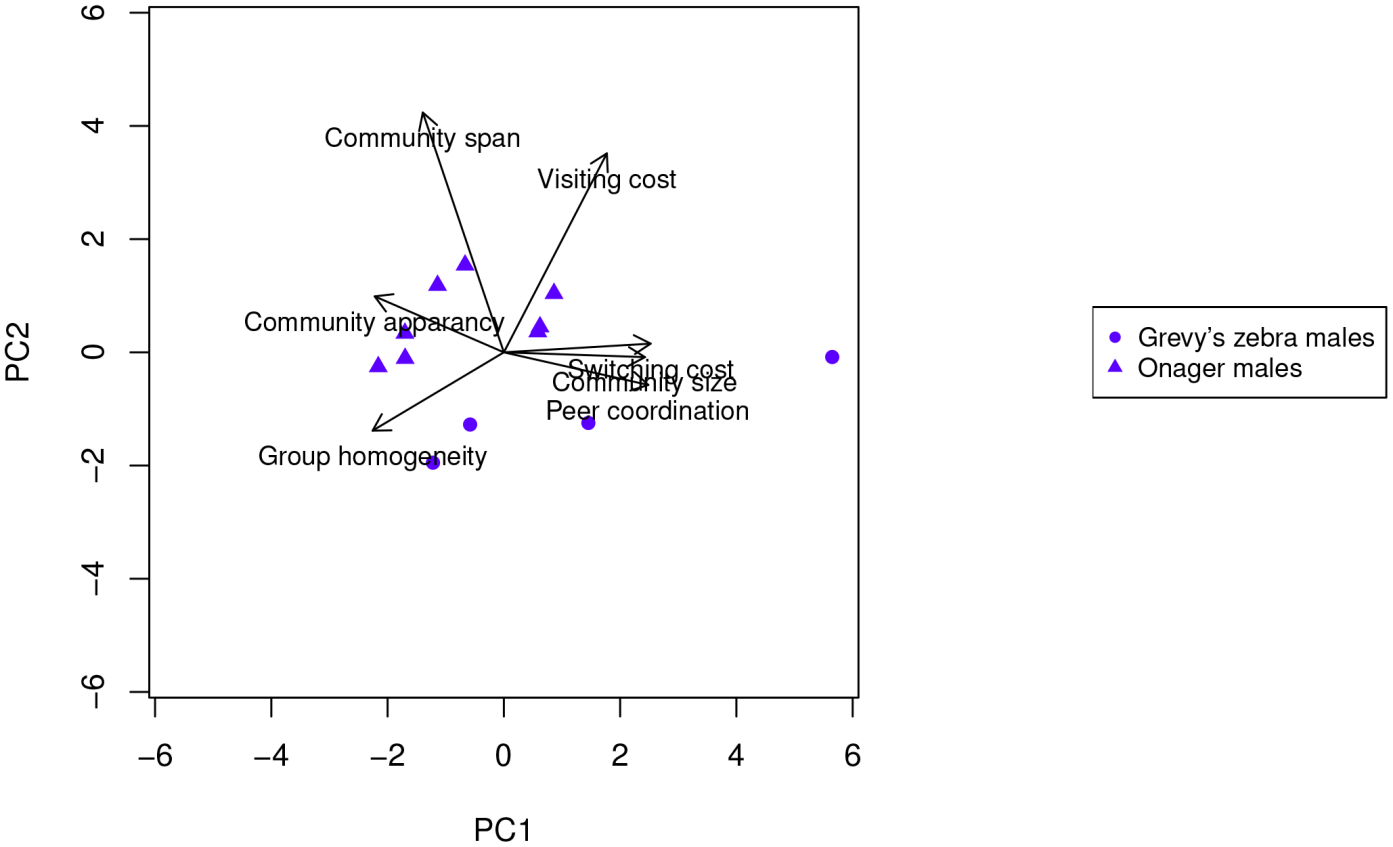
Projection onto the first two principle components of the dynamic communities metrics of all the males in both Grevy’s zebra and onagers.

**Fig 8:**
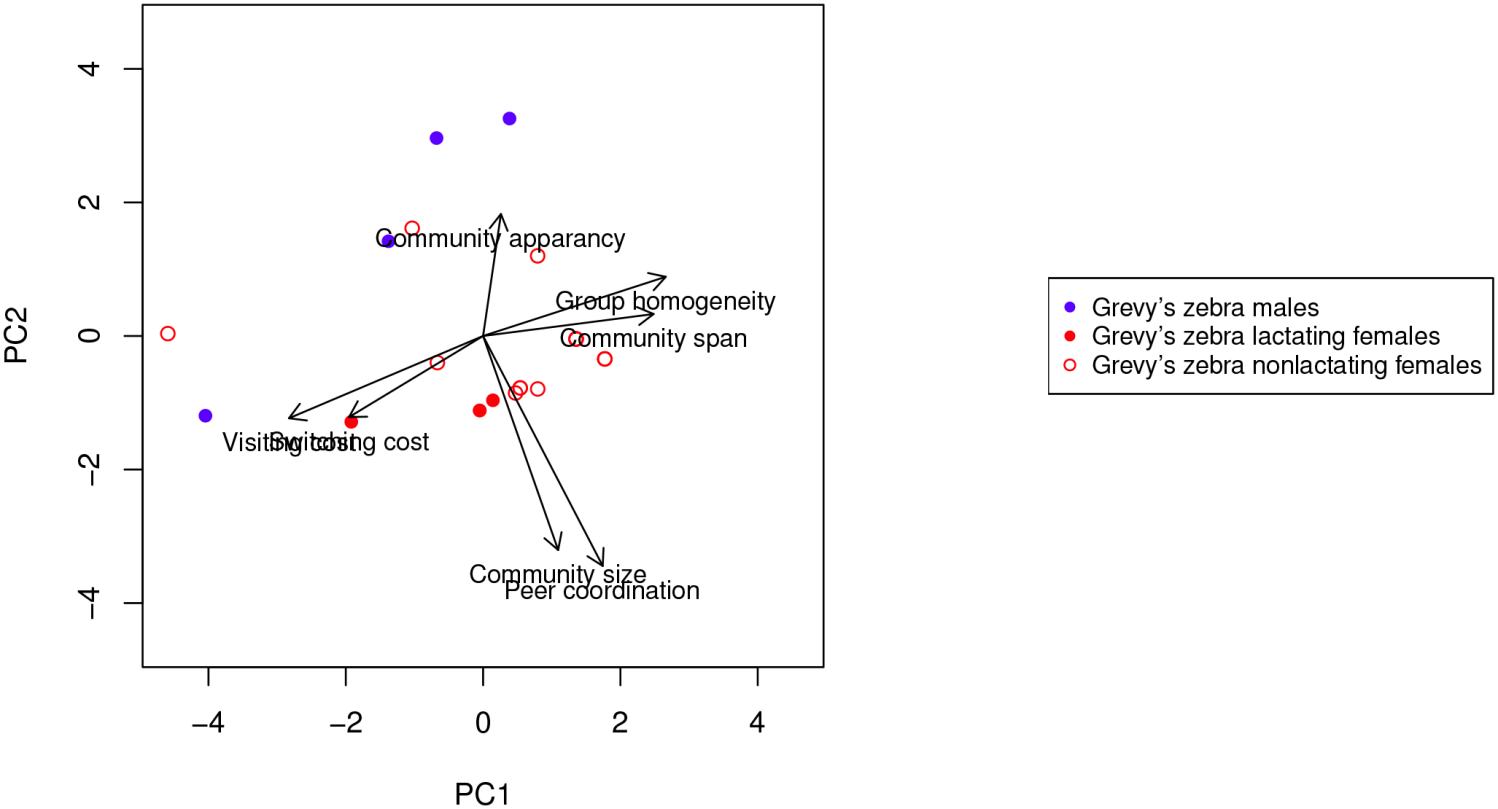
Projection onto the first two principle components of the dynamic communities metrics of all the Grevy’s zebra.

With respect to males (Fig 7), onager males show similar behavior to onager females, while Grevy’s zebras do not (t-test PC1 ratio 1.6, *p* = 0.15, PC2 ratio 4.12, *p* = 0.002). Onager males have a lower switching tendency and have a longer community stay but still have small (often singleton) communities, as do the onager females. While Grevy’s females show a high degree of peer coordination (whatever they do they do together), Grevy’s zebra males show exactly the opposite tendencies. Thus, while females do whatever they do together, males remain alone and reveal no common characterizing factor (Fig 8). T-test PC1 ratio 1.96, *p* = 0.07, PC2 ratio 3.43, *p* = 0.025.

**Fig 9:**
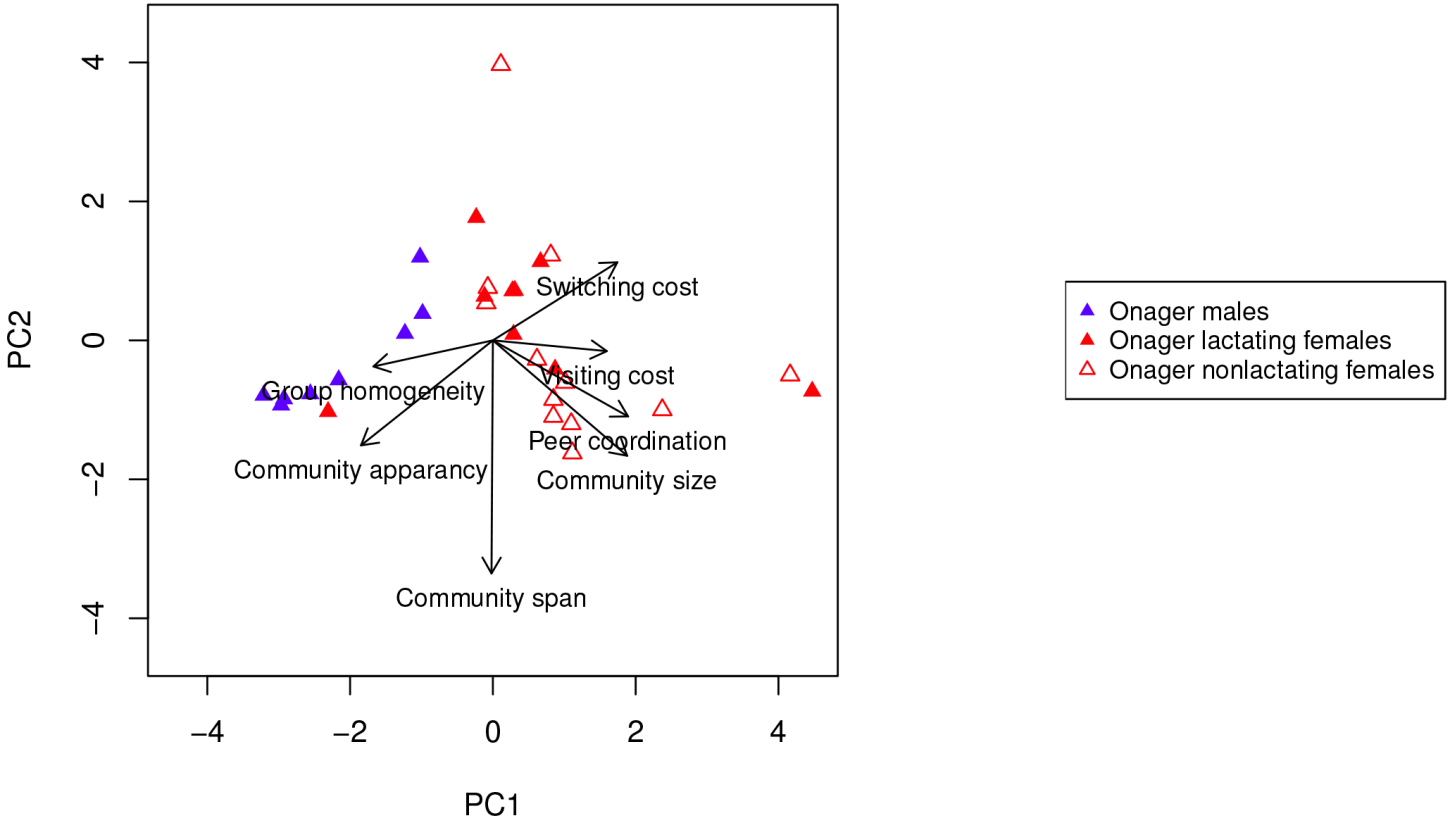
Projection onto the first two principle components of the dynamic communities metrics of all the onagers.

As with the sex difference in characterizing factors for Grevy’s zebra, male and female onagers separate along the factors of peer coordination and community size. T-test PC1 ratio is 5.32, *p* < 0.0001, PC2 ratio 0.78, *p* = 0.44. Fig 9 shows that in this species, too, female communities are more cohesive then those of males. Note, onager males show the longest stay in their singelton communities of all individuals in both species.

## Discussion

The fluid nature of societies poses a challenge for identifying cohesive associations and their underlying causes. Using analysis methods that are explicitly temporal captures this fluidity and exposes the underlying patterns, providing the basis for understanding how and why these patterns emerge and what implications they have on the society structure and function.

In equids, applying dynamic community analysis method revealed large differences between socially similar species of Grevy’s zebra and wild asses, differences that align by sex and reproductive state. These differences only emerged from the temporal analysis, in particular the fact that onager males represent consistent communities of one and the fact that lactating and non-lactating Grevy’s zebra differ in the dynamics of their community affiliations.

Both Grevy’s zebras and onagers are fission-fusion species, but the temporal analysis shows a greater degree of modularity for one. Why should this be so? Evidence emerging from the addition of the temporal component provides new insights for framing hypotheses about the adaptive value and, hence, the functional significance of each equid social network. We conjecture that the persistence of communities comprised of single individuals in onagers, as opposed to the highly peer coordinated, cohesive and large communities of Grevy’s zebras, is driven by differences in the risk of predation and the need to reduce uncertainty associated with the appearance of highly variable resources. As noted previously by Sundaresan *et al.* [3], onagers live in habitats where predators have been extirpated and variance in the availability of water has been reduced, whereas Grevy’s zebras still have to cope with unpredictable movements of lions and uncertainty about drought. Although we engage two selective forces to account for the network differences, we rank predation as the most compelling problem to be solved, much as did Krebs and Dawkins [73] when they framed the ‘Life-dinner’ principle. By remaining in relatively large cohesive groups and switching identity as a unit, we believe Grevy’s zebra minimize the likelihood of being alone on a landscape, a condition where avoiding being eaten is at its lowest [74, 75].

Large relatively cohesive groups, however, should also help reduce the spread of disease while preserving valuable information, thus helping cope with resource acquisition, the other ecological problem needing solving. The dynamic network analysis yielded measures (dynamic cluster coefficient in Table 1) showing that Grevy’s zebra society has a more modular structure than that of onagers. Support for modularity also emerges from the presence of relatively large cohesive coordinated communities of Grevy’s zebras, but not onagers. We conjecture that this modularity is also advantageous because ideas will spread fully within modules, thus preserving the knowledge and information learned from close associates, even if one or more of these individuals disappears from the populations. In contrast, the temporal analysis showed that in onagers communities were small, and although they endured for long periods, they were comprised mostly of single individuals. Consequently, the retention of knowledge is highly dependent on the rate of information transmission and the willingness of any one individual to share or accept that knowledge [76]. The more modular and cohesive dynamic communities of Grevy’s zebra are not as likely dependent on the rate of information transmission and, thus, act as information amplifiers.

For the first time, structures of particular societies can be shown to correlate with the different selective forces generated by particular environmental factors. Although both societies contain the same classes of individuals–males and females, lactating females and non-lactating females–the differences in the societal structures emerge because individuals in these classes act very differently. In general, Grevy’s females show more cohesion by state than do onager females. We have hypothesized that such modularity is likely to reduce the risk of predation and the loss of information. This implies that female relationships lie at the heart of the social dynamics of equid social systems.

Our analysis also shows that when it comes to spreading and retaining ideas, it may be difficult for one structure to maximize both. Conditions fostering spreading may also foster subsequent loss. If retention trumps spreading then networks like those of Grevy’s zebras may be favored. But if the rapid spreading is also of harmful elements such as disease, then community mixing where communities are mostly singletons could be self-cauterizing. As individuals vanish from the population and the network gets sparse, further spreading may be slowed. But such structures would suffer from the rapid loss of individuals with essential information. In environments where resources are predictable such knowledge about location and quality and its retention may not be necessary.

In both onagers and Grevy’s males are not peer coordinated and are in smaller communities then females. In essence they lack community identity since they take on the community membership of the females with which they associate. Computationally this emerges from the CommDy optimization process and sheds little light on the adaptive value of sex differences in community dynamics and motivates further evolutionary and ecological study.

The focus of the network analysis approach taken in the present study is on the persistence of cohesive interactions (communities) over time in two species. The very notions of “persistence over time” and “cohesiveness” are sensitive to the choice of time scale and the number of individuals under consideration. This is not unique to dynamic communities. Reducing the number of nodes will necessarily change the result of the dynamic community analysis (and when only one individual remains, it will always be persistent and cohesive with itself), as it would with any clustering algorithm (of which CommDy is a generalization to temporal clusters). The very definition of clustering problems is sensitive to the identity and relationships among the entities being clustered, particularly in relational clustering problems (see the line of work that started with [77]). A good general discussion on the stability and sensitivity analysis of social networks can be found in [78]. For our study, in the formulation of the dynamic community problem, we assume three sources of noise: (1) missing observations, which we address by using the absence cost, (2) lack of knowledge about the relative magnitude of the costs of switching, visiting, and absence, and (3) the frequency and duration of temporal sampling. We performed sensitivity and stability analysis of our result by varying the relative values of the three costs and we include the results in the supplementary information. Changing the cost values does not change the conclusions and we consider these results highly robust. The issue of temporal sampling is the focus of active research in computational dynamic network analysis and has not been resolved in general [31]. However, in this particular case, we formulate the problem of dynamic communities inference as finding the *most parsimonious* explanation at the temporal scale and for the duration of the sample. As we have shown in [7] and fully proved in [79], the maximum parsimony solution happens to be equal to the maximum likelihood fit of a particular community formation model, which shows that the resulting communities match the temporal scale of sampling. Of course, different community structures may exist at different temporal scales that correspond to different biological phenomena.

In addition to the temporal cohesion, many other aspects of sociality of the two species (and beyond) may be explored. Future studies may focus on the heterogeneity of interactions in various ecological and behavioral contexts, for example, or on leadership and the spread of behavior and information.

Dynamic community analysis of the two equid species showed fundamental differences in their social structure that were correlated with particular ecological conditions. Because of these couplings hypotheses about network structure can be linked to network function. Given the revealing power of this general method, it is likely that when applied to other species in which static (and other biological) analyses have been informative, novel, unexpected but important insights on sociality will emerge. These insights now provide additional information for forming and testing hypotheses as to the selective forces generating these differences and revealing their adaptive value and functional significance.

## Supporting Information

S1 File Stability analysis: exploring the range of relative social cost values. Here we present the dynamic community results for different relative switching, absence, and visiting cost settings. Figs A-C show results for cost settings of switching = 1, absence = 1, visiting = 3. Figs D-F show results for cost settings of switching = 1, absence = 3, visiting = 1. Finally, Figs G-I show results for cost settings of switching = 1, absence = 1, visiting = 1. **Fig A, Inferred dynamic communities of (a) Grevy’s zebra and (b) onagers with costs set to switching = 1, absence = 1, visiting = 3.** **Fig B, Majority superimposed dynamic communities with costs set to switching = 1, absence = 1, visiting = 3.** Superimposed dynamic communities, where each node is colored by the majority color of its dynamic communities ((a) Grevy’s and (b) onagers). **Fig C, Projection onto the first two principle components of the dynamic communities metrics of all the individuals in both Grevy’s zebra and onagers, with costs set to switching = 1, absence = 1, visiting = 3.** **Fig D, Inferred dynamic communities of (a) Grevy’s zebra and (b) onagers with costs set to switching = 1, absence = 3, visiting = 1.** **Fig E, Majority superimposed dynamic communities with costs set to switching = 1, absence = 3, visiting = 1.** Superimposed dynamic communities, where each node is colored by the majority color of its dynamic communities ((a) Grevy’s and (b) onagers). **Fig F, Projection onto the first two principle components of the dynamic communities metrics of all the individuals in both Grevy’s zebra and onagers, with costs set to switching = 1, absence = 3, visiting = 1.** **Fig G, Inferred dynamic communities of (a) Grevy’s zebra and (b) onagers with costs set to switching = 3, absence = 1, visiting = 1.** **Fig H, Majority superimposed dynamic communities with costs set to switching = 3, absence = 1, visiting = 1.** Superimposed dynamic communities, where each node is colored by the majority color of its dynamic communities ((a) Grevy’s and (b) onagers). **Fig I, Projection onto the first two principle components of the dynamic communities metrics of all the individuals in both Grevy’s zebra and onagers, with costs set to switching = 3, absence = 1, visiting = 1.**
(ZIP)
