# Supplementary material for: Similar but Different: Dynamic Social Network Analysis Highlights Fundamental Differences between the Fission-Fusion Societies of Two Equid Species, the Onager and Grevy’s Zebra
Source: PLoS One. 2015 Oct 21;10(10):e0138645. doi: 10.1371/journal.pone.0138645 (PMC4619087; doi:10.1371/journal.pone.0138645)
Supplement: S1 File — Here we present the dynamic community results for different relative switching, absence, and visiting cost settings. Figs A-C show results for cost settings of switching = 1, absence = 1, visiting = 3. Figs D-F show results for cost settings of switching = 1, absence = 3, visiting = 1. Finally, Figs G-I show results for cost settings of switching = 1, absence = 1, visiting = 1. Fig A, Inferred dynamic communities of (a) Grevy’s zebra and (b) onagers with costs set to switching = 1, absence = 1, visiting = 3. Fig B, Majority superimposed dynamic communities with costs set to switching = 1, absence = 1, visiting = 3. Superimposed dynamic communities, where each node is colored by the majority color of its dynamic communities ((a) Grevy’s and (b) onagers). Fig C, Projection onto the first two principle components of the dynamic communities metrics of all the individuals in both Grevy’s zebra and onagers, with costs set to switching = 1, absence = 1, visiting = 3. Fig D, Inferred dynamic communities of (a) Grevy’s zebra and (b) onagers with costs set to switching = 1, absence = 3, visiting = 1. Fig E, Majority superimposed dynamic communities with costs set to switching = 1, absence = 3, visiting = 1. Superimposed dynamic communities, where each node is colored by the majority color of its dynamic communities ((a) Grevy’s and (b) onagers). Fig F, Projection onto the first two principle components of the dynamic communities metrics of all the individuals in both Grevy’s zebra and onagers, with costs set to switching = 1, absence = 3, visiting = 1. Fig G, Inferred dynamic communities of (a) Grevy’s zebra and (b) onagers with costs set to switching = 3, absence = 1, visiting = 1. Fig H, Majority superimposed dynamic communities with costs set to switching = 3, absence = 1, visiting = 1. Superimposed dynamic communities, where each node is colored by the majority color of its dynamic communities ((a) Grevy’s and (b) onagers). Fig I, Projection onto the first two principle c [file pone.0138645.s001.zip › S1File.pdf]

Stability analysis: exploring the range of relative social cost values.

Here we present the dynamic community results for different relative switching, absence, and visiting cost settings. Figs A–C show results for cost settings of switching = 1, absence = 1, visiting = 3. Figs D–F show results for cost settings of switching = 1, absence = 3, visiting = 1. Finally, Figs G–I show results for cost settings of switching = 1, absence = 1, visiting = 1.

Cost settings: switching = 1, absence = 1, visiting = 3

(a) Grevy's zebra

(b) Onagers

Figure A. Inferred dynamic communities of Grevy's zebra and onagers with costs set to switching = 1, absence = 1, visiting = 3.

(a) Grevy's zebra, majority dynamic communities

(b) Onagers, majority dynamic communities

Figure B. Majority superimposed dynamic communities with costs set to switching = 1, absence = 1, visiting = 3. Superimposed dynamic communities, where each node is colored by the majority color of its dynamic communities ((a) Grevy's and (b) onagers).

Figure C. Projection onto the first two principle components of the dynamic communities metrics of all the individuals in both Grevy's zebra and onagers, with costs set to switching = 1, absence = 1, visiting = 3.

Cost settings: switching = 1, absence = 3, visiting = 1

(a) Grevy's zebra

(b) Onagers

Figure D. Inferred dynamic communities of Grevy's zebra and onagers with costs set to switching = 1, absence = 3, visiting = 1.

(a) Grevy's zebra, majority dynamic communities

(b) Onagers, majority dynamic communities

Figure E. Majority superimposed dynamic communities with costs set to switching = 1, absence = 3, visiting = 1. Superimposed dynamic communities, where each node is colored by the majority color of its dynamic communities ((a) Grevy's and (b) onagers).

Figure F. Projection onto the first two principle components of the dynamic communities metrics of all the individuals in both Grevy's zebra and onagers, with costs set to switching = 1, absence = 3, visiting = 1.

Cost settings: switching = 3, absence = 1, visiting = 1

(a) Grevy's zebra

(b) Onagers

Figure G. Inferred dynamic communities of Grevy's zebra and onagers with costs set to switching = 3, absence = 1, visiting = 1.

(a) Grevy's zebra, majority dynamic communities

(b) Onagers, majority dynamic communities

Figure H. Majority superimposed dynamic communities with costs set to switching = 3, absence = 1, visiting = 1. Superimposed dynamic communities, where each node is colored by the majority color of its dynamic communities ((a) Grevy's and (b) onagers).

Figure I. Projection onto the first two principle components of the dynamic communities metrics of all the individuals in both Grevy's zebra and onagers, with costs set to switching = 3, absence = 1, visiting = 1.
